# A survey of emergency department use in patients with cyclic vomiting syndrome

**DOI:** 10.1186/1471-227X-10-4

**Published:** 2010-02-24

**Authors:** Thangam Venkatesan, Sally Tarbell, Kathleen Adams, Jennifer McKanry, Trish Barribeau, Kathleen Beckmann, Walter J Hogan, Nilay Kumar, BUK Li

**Affiliations:** 1 Medical College of Wisconsin, Division of Gastroenterology and Hepatology, 9200, West Wisconsin Avenue, Milwaukee, WI-53226, USA; 2 Medical College of Wisconsin, Division of Pediatric Gastroenterology and Nutrition, 9000 West Wisconsin Avenue Milwaukee, WI 53226, USA; 3 Medical College of Wisconsin, Pediatric Medical Education, 9000 West Wisconsin Avenue, Milwaukee, WI 53226, USA; 4 Children's Research Institute, Children's Hospital of Wisconsin, PO Box 1997 Milwaukee, WI 53201-1997, USA; 5 Medical College of Wisconsin, Division of Pediatric Emergency Medicine, 9000 West Wisconsin Avenue Milwaukee, WI 53226, USA

## Abstract

**Background:**

Cyclic vomiting syndrome (CVS), a chronic disorder characterized by recurrent episodes of vomiting, is frequently unrecognized and is associated with high utilization of emergency department (ED) services.

**Methods:**

A web-based survey was posted on the Cyclic Vomiting Syndrome Association (CVSA) website to assess utilization of ED services in patients with CVS.

**Results:**

Of 251 respondents, 104 (41.4%) were adult CVS patients and 147 (58.6%) were caregivers of pediatric and adult patients. In the adult group, the median number of ED visits for CVS symptoms was 15(range 1 - 200), with a median of 7 ED visits prior to a diagnosis of CVS (range 0 - 150). In the caregiver group, the median number of ED visits was 10 (range 1 - 175) and the median number of ED visits prior to a diagnosis of CVS was 5 (range 0 - 65). CVS was not diagnosed in the ED in 89/104 (93%) adults and 119/147 (93%) patients in the caregiver group. CVS was not recognized in the ED in 84/95 (88%) of adults and 97/122 (80%) of patients in the caregiver group, despite an established diagnosis of CVS.

**Conclusion:**

There is a sub-group of adult and pediatric CVS patients who are high utilizers of ED services and CVS is not recognized in the ED in the majority of patients. Improved efforts to educate ED physicians are indicated to optimize treatment of patients with CVS and to decrease potential overuse of ED services.

## Background

Cyclic vomiting syndrome (CVS) is a chronic idiopathic functional gastrointestinal disorder that is characterized by recurrent, stereotypical, disabling, discrete episodes of intense nausea and vomiting that last a few hours to days, interspersed with varying symptom-free intervals. This disorder is primarily recognized in children, with increasing recognition in adults. The pathophysiology of CVS is unknown, but several theories have been advanced including a dysfunctional brain-gut interaction involving corticotrophin-releasing factor [1], dysregulation of the autonomic nervous system and mitochondrial dysfunction [2-9].

The diagnosis of CVS in adults is based on Rome III criteria: 1) Stereotypical episodes of vomiting regarding onset (acute) and duration (less than 1 week); 2) Three or more discrete episodes in the prior year; and 3)Absence of nausea and vomiting between episodes and absence of metabolic, gastrointestinal, central nervous system structural or biochemical disorders. A personal or family history of migraines is supportive of the diagnosis [10]. The differential diagnosis of CVS includes various gastrointestinal, endocrine, neurological and metabolic problems that can mimic CVS, e.g., hydronephrosis and intestinal malrotation [11-13]. Unfortunately CVS episodes are typically misdiagnosed and there is a 3-8 year delay in diagnosis in adults [14,15] and 2.5 year delay in children [16]. Given the problems with diagnosis of this disorder, it is likely that CVS is more common than currently thought.

In addition, diagnostic uncertainty may lead to suboptimal acute care. Patients with CVS frequently seek care in, or are referred to, the emergency department (ED) for management of acute episodes of vomiting associated with dehydration and electrolyte disturbances. Anecdotally, we believe that familiarity with this disorder among ED personnel is low. The impact of this on acute management and the quality of the patient experience is unclear.

## Aims

The aim of our study was to conduct a survey among patients with CVS about their ED experiences, including recognition of CVS by ED personnel and treatment received in the ED.

## Methods

Two questionnaires were designed for patients with CVS who had visited an ED with symptoms of CVS - one for self-completion by adults with CVS (see additional file 1) and a separate questionnaire for caregivers of patients diagnosed with CVS (see additional file 1). Although intended primarily for pediatric patients, the caregiver survey could be completed by a parent or caregiver of an adult CVS patient.

The survey included demographic information including age, sex and race. Questions included: the total number of ED visits, number of visits before and after recognition of CVS, number of different EDs visited, referral patterns from the ED, and protocols for care. Recognition of CVS and treatment provided in the ED was also assessed. The respondents included all patients who visited the CVSA website and was unlikely to be restricted to a particular geographic area or center.

The surveys were posted on the Web message board of the Cyclic Vomiting Syndrome Association (CVSA) for a period of three months. Patients or caregivers of patients with any prior ED visit related to CVS were invited to participate. The survey was run on http://www.surveymonkey.com. The site and this survey are fully compliant with the Checklist for Reporting Results of Internet E-Surveys (CHERRIES) Web-survey guidelines [17]. Patients and caregivers could voluntarily choose to complete the survey and the study was approved by the Institutional Review Board at our institution.

## Results

There were 251 responses, of which 104 (41.4%) were from adults with CVS and 147 (58.6%) were from caregivers of patients with CVS. The majority of patients in both groups were female and Caucasian (Table 1). Most adult patients 55 (57%) initially presented with CVS symptoms to the ED between the ages of 18-40 years and in the caregiver group, 81 (62%) patients first presented to the ED between the ages of 2-11 years.

**Table 1:** Characteristics of patients with CVS

	Adult group: n = 104		Caregiver group: n = 147	
**Age**	18-24 years	20 (27.7%)	2-5 years	16 (13.7%)
	
	25-39 years	26 (36.1%)	6-11 years	43 (36.8%)
	
	40-65 years	26 (36.1%)	12-17 years	30 (25.6%)
	
			>18 years	28 (23.9%)

**Gender**				

Female	40 (66.7%)		60 (63.8%)	

Male	20 (33.3%)		34 (36.2%)	

**Race**				

White	66 (89.2%)		105 (86.8%)	

Other	8 (10.8%)		15 (12.5%)	

The total number of ED visits reported in this entire population was 2,435 among 251 respondents. Approximately half of ED visits occurred prior to a diagnosis of CVS being made and the recurrent pattern was not recognized (Table 2). In 80% or more of patients, CVS was not recognized in the ER both before and even after the diagnosis was established by a physician elsewhere (Table 2).

**Table 2:** Characteristics of ER visits in patients with CVS

	Adults: n = 104	Caregiver group: n = 147
Number of ER visits per patient with CVS (Median, Range)	15 (1-200)	10 (1-175)

Number of ER visits per patient prior to a diagnosis of CVS being made (Median, Range)	7 (1-150)	5 (0-65)

Diagnosis NOT made in the ER	89(93%)	119(93)%

Diagnosis NOT recognized by the ER in patients with an established diagnosis of CVS	84(88%)	97(80%)

Number of different ERs visited (Mean ± SD)	4.69 ± 4.72	2.6 ± 2.42

ED = emergency department

A minority of patients in the adult group, 31 (32.3%), and 58 (41.7%) in the caregiver group, had protocols for the care of CVS from their treating physician that the patients brought with them to the ED. These protocols consisted of specific instructions regarding management of acute episodes of CVS in the ED by the primary physician/specialist. Among patients who presented to the ED with a protocol for emergency management of CVS, (25/31)81% of adults and (45/58) 80% of patients in the caregiver group had the protocol completely or partially followed.

**Figure 1:**
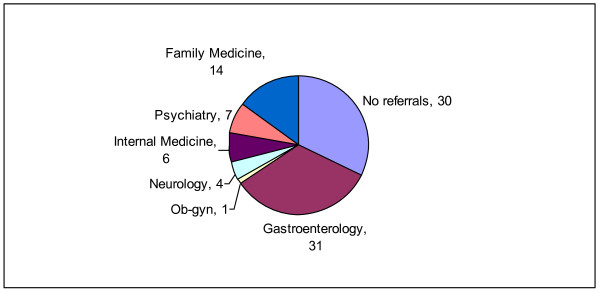
**Emergency room referral patterns of adult patients with cyclic vomiting syndrome**. This figure shows the actual numbers of patients (n = 93) who were either referred or not referred to other specialists; Ob-Gyn = Obstetrics and Gynecology.

**Figure 2:**
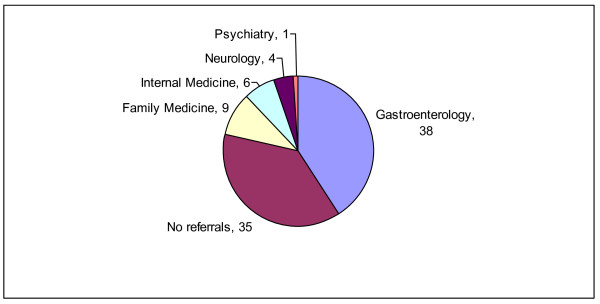
**Emergency department referral patterns of patients in the caregiver group with cyclic vomiting syndrome**. This figure shows the actual numbers of patients (n = 93) who were either referred or not referred to other specialists.

The large majority of patients in both groups - 87/104(84%) of adults and 109/147(74%) of patients in the caregiver group - usually received intravenous fluids as standard care in the ED. Of patients who responded to the question about referral from the ED, approximately a third of the patients in each of the groups who were seen in the ED for CVS symptoms were not referred to specialists for further evaluation of their symptoms (Figures 1 and 2).

## Discussion

This study found that patients with CVS reported that the cause of their symptoms was frequently unrecognized or misattributed in the ED, even among patients with an established diagnosis. The differences in the number of ED visits before diagnosis in children and adults are likely a reflection of the awareness of CVS amongst pediatricians and adult physicians. Though CVS was first described in children in the 19^th ^century, it still remains largely unrecognized in adults despite increasing evidence to the contrary. This is likely due to inadequate knowledge and understanding about the disease and the relative paucity of literature on this disorder especially amongst adult physicians.

Half of the ED visits in our study population occurred prior to the diagnosis; under-recognition likely contributed to this significant delay in diagnosis, as individual episodes may have been attributed to acute viral illnesses or other causes. Delay in making the correct diagnosis results in a lack of preventive care, and may lead to unnecessary interventions, both diagnostic (e.g., endoscopy) and therapeutic (e.g., cholecystectomy) in both adults and children [15].

Even though CVS was not recognized by ED personnel even when patients already bore the diagnosis, the vast majority of all CVS patients received intravenous fluids, an appropriate intervention in patients with dehydration from vomiting of any cause. However, we cannot tell from our results whether dextrose-containing fluids were used or not; this may be important since dextrose-containing intravenous fluids may be therapeutic in the management of acute CVS episodes [11]. It is also encouraging that 80% of patients who presented to the ED with a protocol for acute management of CVS had their protocols followed. Unfortunately only a minority of patients had such care protocols from their physicians. This should prompt physicians who take care of CVS patients to collaborate with ED physicians in establishing individualized protocols for acute management of CVS episodes.

Our patients with CVS had a substantial number of ED visits with high rates of utilization of ED services. In addition to generating an enormous number of ED visits, these patients visited at least four separate EDs on average and this may be an attempt by the patient to seek out effective medical care. Despite repeated visits, the majority of patients in this study were not referred to gastroenterologists.

Inappropriate referrals or non-referrals can lead to further ED visits and also a significant delay in the institution of preventive therapy. The therapy of this disease is very similar to migraine headaches and includes preventive therapies (e.g. amitriptyline), abortive therapy with triptans and supportive strategies (intravenous hydration and sedation) [18,19]. It has been shown that establishing a diagnosis and providing appropriate treatment has a good response in patients with CVS [15].

The economic impact of individual ED visits is staggering and data from our institution indicate that there were 131 visits for CVS in the year 2008. With the cost of a single ED visit being US $2880, the cost of ED management of CVS in our hospital alone would amounts to US $ 377,000 in one year. For cost-effective care it is crucial that steps be taken to address this issue including education about CVS amongst ED personnel and the medical community and further research on newer therapies for CVS. Further efforts in this regard will not only alleviate patient suffering but can potentially transform into saving of hundreds of thousands of dollars.

There are several important limitations of this study. First, all data are self-reported, and therefore subject to recall bias. While such information is reflective of the patient experience, details may not be completely accurate. For example, it is possible that ED personnel were aware of the diagnosis of CVS but may not have communicated this understanding to the patient or caregiver. Respondents, who were identified through the CVSA Web site, may be a select group of patients who are more knowledgeable about CVS, or who have had negative experiences prompting their participation. CVSA members could also be more likely to have a more severe course than others with CVS, although, arguably it is this very subset of patients that needs to be targeted as they utilize enormous health care resources. It is even possible that some respondents do not actually have CVS, but we believe this is unlikely as non-CVS patients would have little incentive to visit the CVSA Web site and participate in the survey.

Also, since this survey only included patients with CVS who had visited an ED, we were unable to ascertain what proportion of CVS patients use the ED or the factors that lead to frequent ED use among patients with CVS. However in the author's own cohort of over a hundred patients with CVS, 13% of patients presented to the ED > 12 times a year (unpublished data). In an effort to protect the personal health information of these patients we did not attempt to obtain geographic location. We are unable to comment about other factors that may be important with regard to ED use among CVS patients such as seasonality or whether these patients were cared for in academic or non-academic centers.

## Conclusions

We conclude that the experience of CVS patients with acute episodes treated in the ED is suboptimal, with delays in recognition and referral, and infrequent use of patient-specific treatment protocols.

Because patients with CVS often present to the ED during acute episodes, ED providers should be familiar with their potential role in this condition: consideration of CVS as a diagnosis in any patient with a history of repeated high-intensity vomiting episodes; supportive care with hydration, dextrose containing fluids, and anti-emetic therapy; and initiation of appropriate referrals from the ED to gastroenterologists or specialists with expertise in this disorder. Care for CVS patients may be improved through education of emergency physicians and staff about this condition and its management.

## Abbreviations

CVS: cyclic vomiting syndrome; ED: emergency department; CVSA: Cyclic Vomiting Syndrome Association

## Competing interests

The authors declare that they have no competing interests.

## Authors' contributions

TV: Study concept and design, Acquisition of the data, Analysis and interpretation of the data, Drafting of the manuscript, Critical revision of the manuscript. ST: Study concept and design, Drafting of the manuscript, Critical revision of the manuscript, Study supervision. TB, JM, KB and KA: Study concept and design, Critical revision of the manuscript, Drafting of the manuscript. WJH: Study concept and design, Critical revision of the manuscript. NK: Study design, Critical revision of the manuscript. BL:Study concept and design, Administrative, technical, or material support, Critical revision of the manuscript. All authors also read and approved the final version of the manuscript.

## Pre-publication history

The pre-publication history for this paper can be accessed here:

http://www.biomedcentral.com/1471-227X/10/4/prepub

## Supplementary Material

Additional file 1 **Web survey for patients and caregivers of patients with CVS**. Original web survey used to gather data from patients with CVS. Click here for file
