# Supplementary material for: A survey of emergency department use in patients with cyclic vomiting syndrome
Source: BMC Emerg Med. 2010 Feb 24;10:4. doi: 10.1186/1471-227X-10-4 (PMC2841069; doi:10.1186/1471-227X-10-4)
Supplement: Additional file 1 — Web survey for patients and caregivers of patients with CVS. Original web survey used to gather data from patients with CVS. [file 1471-227X-10-4-S1.PDF]

1. Cyclic Vomiting Syndrome

You are invited to participate in this survey if you have ever visited an emergency room for the care of cyclic vomiting syndrome (CVS) either as a patient or as a caregiver to a patient. This survey is being done to assess the familiarity of emergency room personnel with CVS and your experience in the emergency room.

This study is being undertaken to collect preliminary data to apply for an educational grant that will serve to disseminate information about CVS to emergency room personnel.

All information will be completely anonymous.

2.

* 1. Please check the category that fits you

- ◯ I am a parent/caregiver of a patient with CVS
- ◯ I am a patient with CVS

3.

* 1. Were you diagnosed with CVS by a physician?

- ◯ Yes
- ◯ No

* 2. At what age did CVS episodes begin?

[▾]

* 3. At what age did you first go to the emergency room for care of CVS?

- ◯ 0-1 years
- ◯ 2-5 years
- ◯ 6-11 years
- ◯ 12-17 years
- ◯ 18-25 year
- ◯ 25-40 years
- ◯ 40 and older

* 4. In TOTAL, how many times have you gone to the emergency room with CVS symptoms?

[ ]

* 5. In TOTAL, how many different emergency rooms have you been to with symptoms of CVS?

[ ]

* 6. How many times TOTAL did you visit the emergency room with CVS symptoms BEFORE the diagnosis of CVS was made?

* 7. Was the diagnosis of CVS made in the emergency room?

- Yes
- No

* 8. If you had the diagnosis of CVS before going to the emergency room, was the diagnosis recognized by the emergency room personnel?

- Yes
- No

* 9. Do you have a written protocol for the care of CVS episodes from your physician who treats CVS?

- Yes
- No

4.

* 1. Was your child diagnosed with CVS by a physician?

- Yes
- No

* 2. At what age did your child's CVS episodes begin?

* 3. At what age did your child first go to the emergency room for care of CVS?

- 0-1 years
- 2-5 years
- 6-11 years
- 12-17 years
- 18 and older

* 4. In TOTAL, how many times has your child patient gone to the emergency room with CVS symptoms?

* 5. In TOTAL, how many different emergency rooms has your child been to with symptoms of CVS?

* 6. How many times TOTAL did your child visit the emergency room with CVS symptoms before the diagnosis of CVS was made?

* 7. Was the diagnosis of CVS made in the emergency room?

- Yes
- No

* 8. If your child had the diagnosis of CVS before going to the emergency room, was the diagnosis recognized by the emergency room personnel?

- Yes
- No

* 9. Does your child have a written protocol for the care of CVS episodes from your physician who treats CVS?

- Yes
- No

5.

* 1. Was the protocol followed by the emergency room staff?

- completely
- partially
- not at all

6.

* 1. Was the protocol followed by the emergency room staff?

- completely
- partially
- not at all

7.

* 1. Does your child have an order from the primary physician for a direct admittance to the hospital rather than going through the emergency room?

- ◯ Yes
- ◯ No

8.

* 1. Do you have an order from the primary physician for a direct admittance to the hospital rather than going through the emergency room?

- ◯ Yes
- ◯ No

9.

* 1. How often is the direct admit order recognized?

- ◯ always
- ◯ sometimes
- ◯ never

10.

* 1. How often is the direct admit order recognized?

- ◯ always
- ◯ sometimes
- ◯ never

11.

* 1. How often has your child received intravenous fluids (IV fluids) in the emergency room?

- ◯ Never
- ◯ Rarely
- ◯ Usually
- ◯ Always

* 2. From the time of entry in the emergency room, about how long is the usual wait before intravenous fluids (IV fluids) are started?

- ◯ 1 hour or less
- ◯ 2-3 hours
- ◯ 3-4 hours
- ◯ 4-5 hours
- ◯ 5 hours or more
- ◯ Have never received IV fluids

* 3. During any emergency room visit, was the patient referred for followup care?

- Yes
- No

12.

* 1. How often do you receive intravenous fluids (IV fluids) in the emergency room?

- Never
- Rarely
- Usually
- Always

* 2. From the time of entry in the emergency room, about how long is the usual wait before intravenous fluids (IV fluids) are started?

- 1 hour or less
- 2-3 hours
- 3-4 hours
- 4-5 hours
- 5 hours or more
- Have never received IV fluids

* 3. During any emergency room visit, were you referred for followup care?

- Yes
- No

13.

* 1. Where was the patient referred?

- Pediatrician
- Family medicine
- Internal medicine
- Gastroenterology
- Neurology
- Psychology/Psychiatry
- Social work

Other (please specify)

14.

* 1. Where were you referred? Choose as many as applicable

- [ ] Family medicine
- [ ] Internal medicine
- [ ] Gastroenterology
- [ ] Neurology
- [ ] Psychology/Psychiatry
- [ ] Social work

Other (please specify)

15.

* 1. What emergency room have you used most of the time?

- ○ Children’s hospital emergency room
- ○ General emergency room
- ○ Both

* 2. What would have helped you or your child most during your visits to the emergency room?

* 3. Please characterize the most frequent attitude you felt in the emergency room staff about your child's care.

- ○ positive
- ○ neutral
- ○ questioning
- ○ negative

Other (please specify)

* 4. How many school days were missed in the year when the patient was having the most episodes?

- ○ 0-10
- ○ 11-20
- ○ 21-30
- ○ More than 30

* 5. Has the parent/caregiver needed to give up employment in order to care for the patient?

- ◯ Yes
- ◯ No

* 6. Has there been disruption in the family unit (separation or divorce) that has been influenced by the illness?

- ◯ Yes
- ◯ No

16.

* 1. What would have helped you most during your visits to the emergency room?

____

* 2. Please characterize the most frequent attitude you felt in the emergency room staff about your care.

- ◯ positive
- ◯ neutral
- ◯ questioning
- ◯ negative

Other (please specify)

____

* 3. How many work days were missed in the year when you were having the most episodes?

- ◯ 0-10
- ◯ 11-20
- ◯ 21-30
- ◯ More than 30

* 4. Which of the following have happened to you as a result of CVS? (Check all that apply)

- [ ] Loss of employment
- [ ] Loss of significant other
- [ ] Delay in higher education
- [ ] Ending of higher education before completion
- [ ] Need for disability designation

* 5. Has substance abuse been a potential problem due to medications needed to help control CVS?

- ◯ Yes
- ◯ no

17.

* 1. What emergency room have you used most of the time?

- [ ] Children's hospital emergency room
- [ ] General emergency room
- [ ] Both

* 2. If you had CVS as a child and have CVS as an adult, have you noted a change in attitude of emergency room personnel towards you?

- ◯ Yes
- ◯ No

18.

* 1. What is the change in attitude of emergency room personnel towards you?

- ◯ Positive
- ◯ Negative

19.

* 1. Have you applied for disability benefits?

- ◯ Yes
- ◯ No

20.

* 1. Have you been awarded benefits?

- Yes
- No
- Pending

21.

1. What is the current age of your child?

- 0-1 years
- 2-5 years
- 6-11 years
- 12-17 years
- 18 and older

2. What is your child's gender?

- Male
- Female

3. Which racial/ethnic group do you belong to?

- Native American/Alaskan Native
- Asian
- African-American
- Hispanic or Latino
- Native Hawaiian or Other Pacific Islander
- White
- Do not wish to answer

Other (please specify)

4. Where do you live?

- Rural < 10,000 population
- Suburban 10,000-79,999
- Urban >80,000
- Don't clearly know

22.

1. What is your current age?

2. What is your gender?

- ◯ Male
- ◯ Female

3. Which racial/ethnic group do you belong to?

- ◯ Native American/Alaskan Native
- ◯ Asian
- ◯ African-American
- ◯ Hispanic or Latino
- ◯ Native Hawaiian or Other Pacific Islander
- ◯ White
- ◯ Do not wish to answer

Other (please specify)

4. Where do you live?

- ◯ Rural < 10,000 population
- ◯ Suburban 10,000-79,999
- ◯ Urban >80,000
- ◯ Don't clearly know

23. Conclusion

Thank you for completing the survey
